# Elevated serum chromogranin A levels are independently associated with metabolic and inflammatory disturbances in polycystic ovary syndrome

**DOI:** 10.3389/fendo.2025.1682910

**Published:** 2025-12-17

**Authors:** Pınar Alarslan, Mehmet Doruk

**Affiliations:** 1 Department of Endocrine and Metabolic Diseases, Istanbul Aydin University, Medical Park Florya Hospital, Istanbul, Türkiye; 2 Department of Endocrine and Metabolic Diseases, İzmir Bozkaya Research and Education Hospital, Bahar, İzmir, Türkiye

**Keywords:** PCOS, chromogranin A, insulin resistance, inflammation, metabolic dysfunction

## Abstract

**Introduction:**

Polycystic ovary syndrome (PCOS) is a multifactorial endocrine disorder characterized by hyperandrogenism, ovulatory dysfunction, and polycystic ovarian morphology, frequently accompanied by insulin resistance, inflammation, and other metabolic disturbances. Chromogranin A (CgA), a glycoprotein secreted by neuroendocrine cells, has been linked to metabolic regulation and inflammatory processes, but its role in PCOS remains unexplored. This study aimed to evaluate serum CgA levels in women with PCOS and their association with metabolic, hormonal, and inflammatory parameters.

**Methods:**

This case-control study included 75 women with PCOS, diagnosed according to the Rotterdam criteria, and 75 age-matched healthy controls, recruited between January and April 2021 at Istanbul Aydın University Medical Park Florya Hospital. Serum CgA levels were measured using ELISA. Clinical parameters and biochemical markers, including glucose, insulin, HOMA-IR, lipid profile, sex hormones, SHBG, and hs-CRP, were assessed. Correlation, multivariate linear regression.

**Results:**

Mean serum CgA levels were significantly higher in the PCOS group than in controls (934.68 ± 256.27 vs. 642.27 ± 197.63 pg/mL, p < 0.001). PCOS patients also had higher glucose, insulin, HOMA-IR, triglycerides, hs-CRP, LH, total testosterone, FAI, and DHEA-S, with lower HDL-C and SHBG (all p < 0.05). CgA correlated positively with BMI, glucose, insulin, HOMA-IR, and hs-CRP, and remained independently associated with BMI, HOMA-IR, and hs-CRP in multivariate analysis. In multivariable models, CgA remained independently associated with BMI, HOMA-IR, and hs-CRP. These findings suggest that CgA reflects the metabolic–inflammatory milieu accompanying PCOS rather than indicating PCOS per se.

**Discussion:**

Elevated CgA levels in PCOS are independently associated with obesity, insulin resistance, and low-grade inflammation. CgA may serve as a novel biomarker for metabolic risk stratification in PCOS, warranting validation in larger, prospective studies.

## 1 Introduction

Polycystic ovary syndrome (PCOS) is the most common endocrine disorder among women of reproductive age, affecting up to 15% of premenopausal women worldwide (1). It is characterized by hyperandrogenism, menstrual irregularities, and polycystic ovarian morphology (2). The etiology is multifactorial, encompassing reproductive, dermatological, and metabolic abnormalities (3).

PCOS is frequently accompanied by insulin resistance, obesity, dyslipidemia, and chronic low-grade inflammation, increasing the risk of type 2 diabetes mellitus, cardiovascular disease, and infertility (4). Despite extensive research, the mechanisms underlying these metabolic alterations remain unclear, involving complex genetic, hormonal, and environmental interactions (5).

Chromogranin A (CgA) is an acidic glycoprotein secreted by neuroendocrine cells that regulates hormone storage and release, including insulin secretion from pancreatic β-cells (5–7). Although primarily used as a biomarker for neuroendocrine tumors, elevated CgA levels have been observed in metabolic and inflammatory disorders such as diabetes, thyroid disease, and cardiovascular conditions (5–8), suggesting its broader role in metabolic regulation and immune signaling.

Evidence on circulating CgA in PCOS is scarce. Given the interplay between neuroendocrine activity, metabolism, and inflammation, evaluating CgA may provide new insight into the metabolic–inflammatory mechanisms of this syndrome.

The aim of the present study is to evaluate serum CgA concentrations in women diagnosed with PCOS and to explore its associations with insulin resistance (assessed by HOMA-IR), systemic inflammation (measured by high-sensitivity C-reactive protein [hs-CRP]), and selected hormonal and metabolic markers. While PCOS is widely recognized for its reproductive implications, increasing attention has been drawn to its broader systemic manifestations, including chronic inflammation, adiposity, and disrupted glucose-insulin homeostasis, which contribute to disease progression and long-term complications (4, 9).

CgA has recently garnered interest not only for its diagnostic utility in neuroendocrine neoplasms, but also for its role in modulating glucose metabolism, lipid regulation, and immune responses (5, 8). However, the relationship between CgA and PCOS has been minimally investigated. Identifying such associations may enhance our understanding of PCOS pathophysiology and position CgA as a novel biomarker for assessing metabolic risk in this population.

## 2 Method

### 2.1 Study design and participants

This was a retrospective analysis of a prospectively maintained biobank. Clinical data were abstracted retrospectively, while stored serum samples (−80 °C) were assayed for CgA in a single batch using ELISA in January and April 2021 at Istanbul Aydın University Medical Park Florya Hospital. A total of 150 female participants were included, consisting of 75 patients diagnosed with polycystic ovary syndrome (PCOS) and 75 age-matched healthy women as controls. All participants were aged between 18 and 35 years. PCOS diagnosis was established based on the Revised Rotterdam Criteria by the American Society for Reproductive Medicine (ASRM), requiring the presence of at least two of the following three conditions: (1) oligo- and/or anovulation, (2) clinical and/or biochemical signs of hyperandrogenism, and (3) polycystic ovarian morphology on ultrasound, defined as ≥12 follicles measuring 2–9 mm in diameter and/or ovarian volume >10 mL in the absence of a dominant follicle or cyst. Transvaginal ultrasound examinations were performed with probes ≥8 MHz; we acknowledge the AE-PCOS/ASRM 2023 recommendations (PCOM threshold ≥20 follicles per ovary) and discuss potential classification impact in the Discussion.

To ensure homogeneity, we included only phenotype A (meeting all three Rotterdam criteria: hyperandrogenism, ovulatory dysfunction, and PCOM). Patients with other conditions that could mimic PCOS, such as congenital adrenal hyperplasia, Cushing’s syndrome, androgen-secreting tumors, and hyperprolactinemia, were systematically excluded.

Biochemical hyperandrogenism was defined as any of the following: serum total testosterone >2.42 nmol/L, dehydroepiandrosterone sulfate (DHEA-S) >248 µg/dL, or a free androgen index (FAI) >5%. Clinical hyperandrogenism (hirsutism) was evaluated using the Ferriman-Gallwey (FG) score, with a threshold of ≥8 indicating significant hirsutism. The same clinician performed all FG assessments to reduce inter-observer variability.

#### 2.1.1 Exclusion criteria

Exclusion criteria for both groups included:

- Pregnancy, lactation, or galactorrhea
- Acute or chronic infections, autoimmune diseases, chronic inflammatory conditions
- Thyroid dysfunction, systemic diseases affecting insulin sensitivity (e.g., impaired glucose tolerance, diabetes mellitus types 1 and 2, gestational diabetes)
- Cardiovascular diseases (e.g., hypertension, coronary artery disease, heart failure), liver or renal failure, malignancy
- Smoking or excessive alcohol consumption
- Use of medications affecting insulin sensitivity, lipid levels, sex steroids, or blood pressure within the past six months
- Use of proton pump inhibitors or H2-receptor blockers within 2 weeks prior to sampling

Participants with familial hyperlipidemia were also excluded.

#### 2.1.2 Ethical approval

The study protocol was approved by the Non-Interventional Clinical Research Ethics Committee of Istanbul Aydın University (Approval No: GOKAEK 050.06.04/447, Date: 11.04.2021). Written informed consent was obtained from all participants prior to data collection.

### 2.2 Biochemical measurements

Blood samples were collected from the antecubital vein between 08:00 and 09:00 AM after an overnight fast of at least 12 hours, during the early follicular phase of the menstrual cycle (days 3–5) or following progesterone-induced withdrawal bleeding in cases of amenorrhea. Serum glucose, total cholesterol (TC), triglycerides (TG), and high-sensitivity C-reactive protein (hs-CRP) were measured using photometric methods on an Olympus AU2700 autoanalyzer (Beckman Coulter Inc., CA, USA), and low-density lipoprotein cholesterol (LDL-C) was calculated using the Friedewald formula. Glycated hemoglobin (HbA1c) was determined by high-performance liquid chromatography with the Adams HA-8160 HbA1c analyzer (Menarini, Italy). Serum follicle-stimulating hormone (FSH), luteinizing hormone (LH), progesterone, and estradiol levels were analyzed using chemiluminescent immunoassay (CMIA) on the UniCel DxI 800 analyzer (Beckman Coulter), while insulin concentrations were assessed with CMIA on the Abbott Architect i2000 analyzer. Insulin resistance was calculated using the homeostasis model assessment (HOMA-IR) formula: HOMA-IR = (fasting glucose [mg/dL] × fasting insulin [µIU/mL])/405. Androgen markers, including total testosterone, sex hormone-binding globulin (SHBG), and dehydroepiandrosterone sulfate (DHEA-S), were measured by radioimmunoassay, and the free androgen index (FAI) was calculated as FAI (%) = (total testosterone [nmol/L]/SHBG [nmol/L]) × 100. Serum chromogranin A (CgA) concentrations were determined using a commercially available enzyme-linked immunosorbent assay (ELISA) kit (R&D Systems, Minneapolis, MN, USA) with a detection range of 62.5–4000 pg/mL and intra-/inter-assay coefficients of variation below 10%.The assay’s limit of detection was ~20 pg/mL; all CgA measurements were performed in a single batch to minimize between-run variability, and samples were stored at −80 °C until analysis. Reference ranges for all analytes (including CgA) are provided in the table footnotes. Serum androstenedione was not measured and is acknowledged as a study limitation.

### 2.3 Statistical analysis

Data analyses were conducted using SPSS software version 21.0 (SPSS Inc., Chicago, IL, USA). The normality of data distribution was evaluated using the Kolmogorov–Smirnov test. Where appropriate, variables were log-transformed to approximate normality. Continuous variables were presented as mean ± standard deviation (SD), while categorical variables were expressed as numbers and percentages. For group comparisons, the independent-samples t-test was applied to normally distributed continuous variables, and chi-square tests were used for categorical variables. Pearson’s correlation coefficients were calculated to assess the relationships between serum chromogranin A (CgA) levels and other clinical or biochemical parameters. Multiple linear regression analysis was performed to identify independent predictors of serum CgA concentrations with adjustments made for potential confounders, including body mass index (BMI), homeostasis model assessment of insulin resistance (HOMA-IR), and high-sensitivity C-reactive protein (hs-CRP). Normality was assessed (Kolmogorov–Smirnov) and log-transformation applied where appropriate. Between-group differences are accompanied by effect sizes (Cohen’s *d*) with 95% CIs. For non-primary multiple comparisons, Benjamini–Hochberg false discovery rate (FDR) control was applied. Multicollinearity was quantified using the *Variance Inflation Factor (VIF)* (near 1, negligible; >5, moderate; ≥10, severe); all predictors had VIF<2.0. The *a priori* multivariable linear model included BMI (adiposity), HOMA-IR (insulin resistance), and hs-CRP (low-grade inflammation) as biologically grounded axes. Glucose and insulin were not co-modeled with HOMA-IR to avoid redundancy and variance inflation. Linearity (Box–Tidwell), homoscedasticity, and residual normality assumptions were met. A p-value of less than 0.05 was considered statistically significant.

## 3 Results

A total of 75 PCOS patients and 75 healthy participants were included in the study. The mean age was 28.11 ± 4.72 years in patients diagnosed with PCOS and 27.70 ± 4.83 years in healthy subjects (*p =* 0.592). No significant differences for clinical features including body mass index, systolic and diastolic blood pressure were found between the two groups (all, *p >* 0.05) (**Table 1**).

**Table 1:** The demographic and biochemical characteristics of participants.

Variables	PCOS (n=75)	Controls (n=75)	*p* value
Age, years	28.11 ± 4.72	27.70 ± 4.83	0.592
BMI, kg/m^2^	26.41 ± 4.46	27.10 ± 4.53	0.332
SBP, mmHg	109.35 ± 13.21	107.27 ± 11.64	0.292
DBP, mmHg	74.10 ± 6.58	73.61 ± 5.61	0.612
Ferriman-Gallwey score	14.66 ± 2.82	4.26 ± 1.16	<0.001*
Glucose, mg/dl	83.86 ± 8.13	81.59 ± 5.71	0.043*
Insulin, µIU/ml	17.18 ± 6.11	10.72 ± 4.45	<0.001*
HOMA-IR	3.58 ± 1.39	2.15 ± 0.89	<0.001*
HbA1_C_, %	5.27 ± 0.18	5.24 ± 0.19	0.221
Total cholesterol, mg/dl	207.01 ± 33.35	199.92 ± 43.43	0.250
LDL-C, mg/dl	137.30 ± 28.39	129.76 ± 27.47	0.090
HDL-C, mg/dl	41.28 ± 9.87	48.50 ± 10.78	<0.001*
Triglycerides, mg/dl	142.05 ± 32.68	108.28 ± 29.80	<0.001*
hs-CRP, mg/l	1.20 ± 0.55	0.67± 0.19	<0.001*
FSH, mIU/ml	6.84 ± 1.77	7.20 ± 1.88	0.208
LH, mIU/ml	14.16 ± 4.24	8.48 ± 3.04	<0.001*
Progesterone, ng/ml	1.14 ± 0.26	1.19 ± 0.25	0.203
Estradiol, pg/ml	50.37 ± 11.89	48.93 ± 7.99	0.369
Total-testosterone, nmol/l	2.89 ± 0.41	1.68 ± 0.36	<0.001*
SHBG, nmol/l	37.23 ± 11.52	67.90 ± 15.01	<0.001*
FAI, %	8.22 ± 1.72	2.48 ± 0.11	<0.001*
DHEA-S, µg/dl	182.63 ± 71.63	151.43 ± 39.21	0.001*
Chromogranin A, pg/ml	934.68 ± 256.27	642.27 ± 197.63	<0.001*

PCOS, Polycystic ovary syndrome; BMI, Body mass index; DBP, Diastolic blood pressure; SBP, Systolic blood pressure; HbA1c, Glycosylated hemoglobin; HOMA-IR, Homeostasis model assessment of insulin resistance; LDL-C, Low density lipoprotein cholesterol; HDL-C, High density lipoprotein cholesterol; hs-CRP, High sensitivity C-reactive protein; FSH, follicle-stimulating hormone LH, Luteinizing hormone; SHBG, Sex hormone-binding globulin; FAI, Free androgen index; DHEA-S, Dehydroepiandrosterone sulfate. Data were given as mean ± standard deviation. Independent samples t-test was used for comparison. A *p* value of <0.05 was considered significant (*).

Bold values indicate statistically significant results (p < 0.05).

The mean serum CgA levels were found to be high in patients with PCOS (934.68 ± 256.27 pg/mL) compared to controls (642.27 ± 197.63 pg/mL) (*p =* < 0.001). The mean serum levels of glucose, insulin, HOMA-IR, TG, hs-CRP, LH, total testosterone, FAI, DHEA-S were significantly higher in PCOS patients than healthy individuals (all, *p <* 0.05). The mean HDL-C and SHBG levels of PCOS patients were lower in PCOS patients (all, *p <* 0.05). The mean levels of HbA1c, total cholesterol, LDL-C, progesterone, estradiol and FSH were observed similar between the two groups (all, *p >* 0.05) (**Table 1**).

Correlation analyses between participants’ CgA levels and clinical and biochemical features were shown in **Table 2**. CgA levels were positively correlated with BMI, insulin, glucose, HOMA-IR and hs-CRP levels (all, *p <* 0.05). No significant correlations were found between CgA levels and the levels of lipid metabolism markers (TG, total cholesterol, LDL-C, HDL-C) or sex hormone parameters (estradiol, progesterone, FSH, LH, FAI, DHEA-S) (all, *p >* 0.05). Results for the independent association between CgA and other parameters obtained from multiple linear regressions were given in **Table 3**. CgA levels were independently related with BMI, HOMA-IR and hs-CRP.

**Table 2:** Correlation analyses between Chromogranin A levels and participants’ clinical and biochemical characteristics.

Variables	Correlation analysis	
	*r* value	*p* value
Age	0.114	0.132
BMI	0.104	0.032*
Insulin	0.323	0.004*
Glucose	0.264	0.012*
HOMA-IR	0.281	0.009*
hs-CRP	0.116	0.027*
FSH	0.094	0.192
LH	0.067	0.345
Estradiol	0.101	0.132
Progesterone	0.087	0.234
FAI	0.192	0.212
DHEA-S	0.789	0.105
Total cholesterol	0.064	0.301
LDL-C	0.783	0.267
HDL-C	-0.102	0.087
Triglycerides	0.112	0.175

BMI, Body mass index; HOMA-IR, Homeostasis model assessment of insulin resistance; FAI, Free androgen index; hs-CRP, High sensitivity C-reactive protein; FSH, follicle-stimulating hormone LH, Luteinizing hormone; DHEA-S, Dehydroepiandrosterone sulfate; HDL-C, High density lipoprotein cholesterol; LDL-C, Low density lipoprotein cholesterol. Pearson’s correlation analysis was used. r: Pearson’s correlation coefficient. A *p* value of < 0.05 was considered significant (*).

Bold values indicate statistically significant results (p < 0.05).

**Table 3:** Multiple linear regression analysis of correlated variables with Chromogranin A levels in all study population.

Variables	Multiple regression analysis			
	β	95% CI		p value
		Lower	Upper	
BMI	0.096	0.085	0.107	0.024*
HOMA-IR	0.167	0.097	0.237	0.015*
hs-CRP	0.101	0.088	0.114	0.021*

BMI, Body mass index; HOMA-IR, Homeostasis model assessment of insulin resistance; hs-CRP, High sensitivity C-reactive protein. β, Unstandardized regression coefficient; CI, Confidence interval. A *p* value of < 0.05 was considered significant (*).

**Figure 1:**
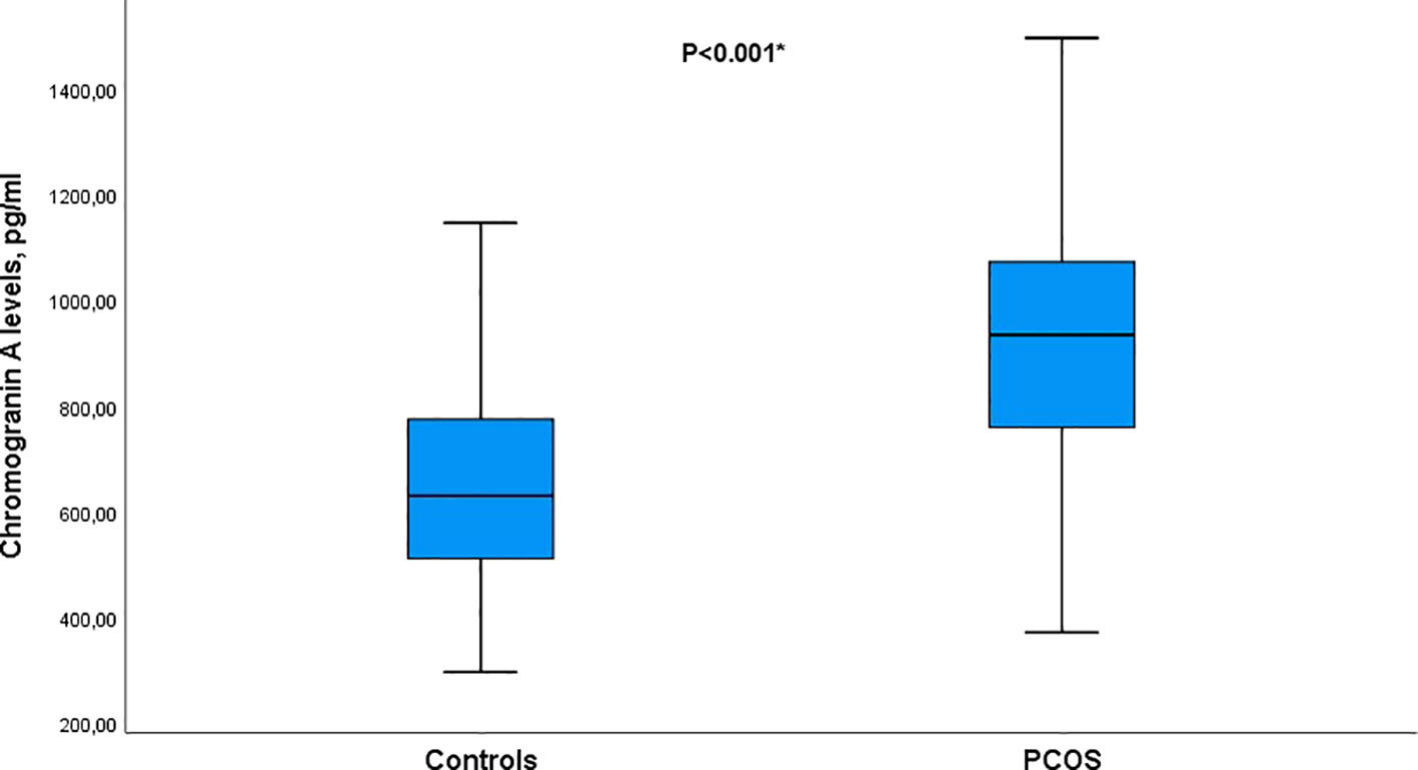
Boxplot of serum Chromogranin A (CgA) levels in women with PCOS and controls. Median CgA levels were significantly higher in the PCOS group compared with controls (*p* < 0.001).

The case processing summary for the boxplot analysis of PCOS and control groups is presented in **Table 4**, **Figure 1**. A total of 150 cases (75 PCOS and 75 control subjects) were analyzed with no missing data. All statistical analyses were performed on complete cases.

**Table 4:** Case processing summary for the Boxplot analysis of PCOS groups.

Variable	Group (PCOS)	Valid (n)	Valid (%)	Missing (n)	Missing (%)	Total (n)	Total (%)
Chromogranin A (CgA), pg/mL	0 (Control)	75	100.0%	0	0.0%	75	100.0%
	1 (PCOS)	75	100.0%	0	0.0%	75	100.0%

## 4 Discussion

In this study, we demonstrated that serum chromogranin A (CgA) levels were significantly elevated in women with polycystic ovary syndrome (PCOS) and showed a positive correlation with body mass index (BMI), insulin, glucose, HOMA-IR, and high-sensitivity C-reactive protein (hs-CRP). Moreover, the associations between CgA and BMI, HOMA-IR, and hs-CRP were found to be independent. Our data also confirmed that patients with PCOS exhibited elevated levels of glucose, insulin, HOMA-IR, triglycerides (TG), hs-CRP, luteinizing hormone (LH), total testosterone, free androgen index (FAI), and dehydroepiandrosterone sulfate (DHEA-S), while levels of high-density lipoprotein cholesterol (HDL-C) and sex hormone-binding globulin (SHBG) were significantly lower. Taken together, these findings support a model in which CgA aligns with the metabolic–inflammatory milieu that frequently accompanies PCOS rather than indicating PCOS per se. This interpretation is consistent with comprehensive evidence showing heightened low-grade inflammation and metabolic dysregulation in PCOS (10–12) and with causal inference work suggesting that inflammatory protein pathways may influence PCOS risk through metabolically relevant intermediates (13).

PCOS is a complex endocrine and gynecological disorder characterized by hormonal dysregulation, with implications for metabolic, dermatological, reproductive, and psychological health (8, 9). Although the precise molecular mechanisms remain unclear, hyperandrogenism and insulin resistance are considered key features in the pathophysiology of PCOS (2). Hyperandrogenism has been implicated in disrupting metabolic homeostasis in PCOS patients (14). Androgen excess may promote adipocyte lipid accumulation, dyslipidemia, and an altered LH/FSH ratio (15). Both obesity and insulin resistance are believed to synergistically stimulate increased ovarian and adrenal androgen production, further elevating circulating testosterone levels (15, 16). The altered LH/FSH ratio may additionally enhance androgen synthesis in theca cells and impair aromatase activity and estrogen production in granulosa cells, creating a feed-forward loop that sustains hyperandrogenism (17). In parallel, contemporary syntheses and patient-level studies underscore that overweight/obesity amplifies insulin resistance, lipotoxicity, and systemic inflammation in PCOS, shaping its metabolic phenotype (10–12) —a context in which a neuroendocrine secretory protein such as CgA could plausibly track aggregate metabolic–inflammatory load.

The resulting endocrine and metabolic disturbances, including androgen excess, insulin resistance, low-grade inflammation, and dyslipidemia, contribute to a variety of PCOS manifestations, such as infertility, increased cardiometabolic risk, mood disorders, and dermatological signs like acne, hirsutism, and androgenic alopecia (14). In line with these mechanisms, our findings of elevated total testosterone, LH, DHEA-S, glucose, insulin, HOMA-IR, TG, hs-CRP, and FAI, as well as decreased HDL-C and SHBG in PCOS women, support the role of hormonal imbalance in PCOS pathogenesis. These findings are consistent with previous literature indicating that impaired glucose tolerance, chronic inflammation, dyslipidemia, and disturbed gonadotropin and androgen levels are commonly observed in PCOS (2, 14–17). Notably, systematic evaluations of inflammatory biomarkers in PCOS (e.g., CRP, IL-6, TNF-α) show consistent elevations compared with controls (10); mechanistic overviews and cohort analyses in overweight PCOS further delineate mitochondrial dysfunction, altered adipokine signaling, and hepatic insulin resistance as central features of the metabolic disorder (11, 12). Our observation that CgA independently associates with HOMA-IR and hs-CRP dovetails with this framework, positioning CgA as a candidate integrative marker at the neuroendocrine–metabolic–immune interface.

Identifying novel endocrine markers that reflect the metabolic disruptions in PCOS may provide insight into the underlying mechanisms and open avenues for new diagnostic or therapeutic strategies. In this regard, Mendelian randomization linking inflammatory proteins to PCOS via serum metabolite pathways provides orthogonal support for an inflammation-to-metabolism axis in PCOS (13) and strengthens the rationale for exploring neuroendocrine secretory products (including CgA and its fragments) as upstream or parallel readouts of these processes.

CgA is a member of the granin family of acidic secretory proteins released by endocrine and neuroendocrine cells in a variety of organs (18). CgA and its biologically active cleavage products—including serpinin, catestatin, vasostatin, and pancreastatin—have been implicated in regulating hormone secretion, cell proliferation, cardiovascular function, glucose and lipid metabolism, immune response, neurotransmitter release, and reproductive function (6, 19). These fragments exhibit diverse, sometimes opposing, physiological effects; for instance, serpinin is pro-adrenergic, while vasostatin I and catestatin exert anti-adrenergic effects (4). Moreover, serpinin functions as an anti-apoptotic agent, whereas vasostatin I promotes apoptosis (20). Owing to these multifaceted roles, CgA and its derivatives have been linked to a wide range of both benign and malignant diseases.

Although serum CgA levels are widely used for diagnosing and monitoring neuroendocrine tumors, their clinical utility is limited by low specificity (21). Elevations of CgA have been reported in gynecologic malignancy (e.g., uterine corpus large-cell neuroendocrine carcinoma and ovarian carcinoma) and may even occur in benign conditions (leiomyoma/endometriosis) (22–25). Thus, our findings are not intended to imply oncologic risk; instead, we contextualize CgA as a nonspecific marker that tracks the metabolic–inflammatory burden in PCOS. Given our metabolic emphasis, oncologic speculations were removed to maintain scope and relevance, and we instead contextualize CgA within cardio-metabolic and inflammatory biology.

Beyond oncological applications, emerging evidence suggests a role for CgA and its cleavage products in several non-malignant conditions, including cardiovascular diseases (e.g., heart failure, myocardial infarction, hypertension), chronic inflammatory states (e.g., severe sepsis, inflammatory bowel disease, rheumatoid arthritis), and metabolic disorders (e.g., diabetes mellitus, metabolic syndrome) (5, 6, 26). CgA is widely distributed in endocrine tissues, including pancreatic β-cells and α-cells that secrete insulin and glucagon, respectively. In CgA knockout mice, obesity and peripheral insulin resistance coexist with improved hepatic insulin sensitivity (27). Pancreastatin has been shown to impair glucose-stimulated insulin secretion and induce insulin resistance (27), whereas catestatin appears to exert anti-obesity effects by enhancing lipid mobilization and leptin signaling (6). These pleiotropic, sometimes bidirectional actions mirror the heterogeneity of PCOS pathophysiology and offer plausible biological routes through which CgA could correlate with both insulin resistance and low-grade inflammation in clinical cohorts (10–12).

The involvement of CgA in metabolic regulation has prompted investigations into its role in metabolic diseases. Kogawa et al. reported significantly higher plasma and salivary CgA levels in patients with type 2 diabetes mellitus (28). To date, only one study has investigated serum CgA levels in PCOS. In that study, Gennarelli et al. assessed the counter-regulatory response to insulin-induced hypoglycemia and found no significant differences in basal or stimulated CgA levels between PCOS patients and healthy controls, suggesting a normal physiological response to hypoglycemia in PCOS (29). Methodological differences likely explain the discrepant findings: our basal, fasting measurements and single-batch ELISA of stored sera probe chronic homeostasis, whereas hypoglycemia-challenge paradigms interrogate acute counter-regulation; in addition, contemporary evidence emphasizes chronic inflammatory signaling and metabolic reprogramming in PCOS (10–13), which aligns more closely with our basal CgA associations.

In contrast to that study, our findings demonstrated that serum CgA levels are significantly elevated in women with PCOS and independently associated with BMI and HOMA-IR. This supports the hypothesis that CgA may contribute to the regulation of metabolic homeostasis and play a role in the development of insulin resistance and obesity. Additionally, the observed correlation between CgA and hs-CRP levels suggests a potential involvement of CgA in low-grade inflammation, a well-documented feature of PCOS. These results are in line with prior studies linking CgA and its derivatives to inflammatory processes (5, 6, 26). Looking forward, two design refinements would strengthen causal interpretation: (i) comparison with metabolically matched non-PCOS controls (matched on BMI, HOMA-IR, and inflammatory profiles) to test whether CgA elevation persists beyond comorbidity, and (ii) integration of unbiased inflammatory/metabolomic panels (guided by Mendelian randomization signals) to localize CgA within specific inflammatory–metabolic pathways (10, 11, 13).

Despite these novel insights, our study has several limitations. First, the sample size was relatively small, which may limit the generalizability of our findings. Second, CgA is a highly heterogeneous antigen with variable post-translational modifications and proteolytic processing, which may affect assay results. Third, serum CgA levels can be influenced by a variety of factors including renal and hepatic dysfunction, pregnancy, intense physical activity, and the use of certain medications such as proton pump inhibitors and histamine-2 receptor antagonists (30). Fourth, androstenedione was not measured, which limits the granularity of our androgen profiling. Finally, variations in assay techniques for CgA measurement, due to a lack of standardization, may contribute to inconsistencies in reported values. Nonetheless, our single-batch approach and predefined modeling reduce analytic noise and focus the interpretation on biologically grounded axes (adiposity, insulin resistance, inflammation).

In conclusion, our study indicates that elevated serum CgA levels are associated with PCOS and reflect underlying metabolic and inflammatory disturbances. By aligning with HOMA-IR and hs-CRP independent of univariable correlates, CgA emerges as a potential integrative marker of the metabolic–inflammatory burden that characterizes many PCOS phenotypes (10–12).CgA may serve as a potential biomarker or pathophysiological mediator in PCOS-related insulin resistance and inflammation. Further prospective studies with larger sample sizes are warranted to validate these findings and elucidate the mechanistic roles of CgA and its fragments in PCOS. Incorporating metabolically matched comparators and pathway-level tools (e.g., targeted proteomics/metabolomics informed by MR) will be essential to determine whether CgA has incremental value over established inflammatory markers in risk stratification (10, 11, 13).

### 4.1 Limitation

The main limitations of this study include the relatively small sample size and the retrospective design, which may limit the generalizability of the findings. Additionally, factors that may influence serum CgA levels—such as unrecognized subclinical conditions, dietary factors, or non-standardized assay variability—could not be completely excluded. The absence of androstenedione measurements and the lack of metabolically matched non-PCOS comparators further limit causal inference.

## 5 Conclusion

This study investigates serum chromogranin A (CgA) levels and their association with endocrine and metabolic features in patients with polycystic ovary syndrome (PCOS). Our findings suggest that CgA may play a significant role in the regulation of metabolic homeostasis and inflammatory processes in PCOS. Elevated serum CgA levels were independently associated with insulin resistance, low-grade inflammation, and obesity, indicating its potential utility as a biomarker for metabolic dysregulation in PCOS. Further large-scale and prospective studies are warranted to confirm these findings and explore the diagnostic or therapeutic value of CgA in this population.

## Data Availability

The raw data supporting the conclusions of this article will be made available by the authors, without undue reservation.
